# Glycemic Control and Insulin Requirement According to Enteral Formula Type in Critically Ill Patients with Type 2 Diabetes: A Retrospective Comparative Study

**DOI:** 10.3390/nu18101615

**Published:** 2026-05-20

**Authors:** Serpil Ekin, Derful Gülen, İlkay Ceylan, Buket Özyaprak, Kamer Kılınç, Aslıhan Öztürk

**Affiliations:** 1 Department of Anesthesiology and Reanimation, Bursa Yuksek Ihtisas Training and Research Hospital, University of Health Sciences, Bursa 16310, Turkey; 2 Department of Nutrition and Dietetics, Bursa Yuksek Ihtisas Training and Research Hospital, University of Health Sciences, Bursa 16310, Turkey

**Keywords:** type 2 diabetes mellitus, critical illness, enteral nutrition, standard enteral formula, diabetes-specific enteral formula, glycemic control, insulin requirement, cost

## Abstract

**Background/Objectives**: This study evaluated whether a low-carbohydrate diabetes-specific enteral formula improves glycemic control and insulin requirement compared with a standard enteral formula in critically ill patients with type 2 diabetes mellitus (T2DM) under pandemic-related product accessibility constraints. **Methods**: This retrospective observational study included adult ICU patients with T2DM receiving enteral nutrition between August 2021 and August 2023. Patients were grouped according to enteral formula type as standard enteral formula or diabetes-specific enteral formula. All patients received continuous nasogastric enteral feeding according to routine ICU practice. Glycemic control was managed using intravenous insulin infusion protocols. One hundred eligible patients were analyzed. **Results**: Fifty patients were included in each group. Baseline characteristics were broadly comparable, although differences in BMI and feeding rate were observed. Mean glucose level, daily insulin requirement, hypoglycemia, hyperglycemia, and glycemic variability were similar between groups (all *p* > 0.05). However, the number and percentage of days within the target glycemic range were higher in the diabetes-specific formula group (both *p* = 0.021). Clinical outcomes were comparable between groups. In multivariable analysis, mean glucose level independently predicted insulin requirement and glycemic variability, whereas formula type did not. Product-related costs were lower in the diabetes-specific formula group (all *p* < 0.001). **Conclusions**: Diabetes-specific enteral formula did not improve mean glucose level or insulin requirement in critically ill patients with T2DM, although it was associated with better maintenance of the target glycemic range and lower product-related costs. Enteral formula choice should therefore be individualized rather than routinely determined by diabetes status alone.

## 1. Introduction

During the COVID-19 pandemic, extraordinary healthcare conditions and supply-chain disruptions restricted access to diabetes-specific enteral formulas at our institution, necessitating the use of standard formulas in some critically ill patients with type 2 diabetes mellitus (T2DM). This circumstance raised a practical clinical question: whether standard enteral formulas could serve as an acceptable alternative when diabetes-specific formulas are unavailable.

Hyperglycemia is common in critically ill patients as part of the stress response, regardless of pre-existing diabetes, and has become a central component of glycemic management in the ICU [1,2]. The metabolic stress response promotes insulin resistance, increased hepatic glucose production, and impaired peripheral glucose utilization, often making glycemic control more difficult in patients with T2DM [3,4,5,6]. Although hyperglycemia has been associated with infection, impaired immune function, prolonged hospitalization, and increased morbidity [7], overly aggressive glucose lowering may also cause hypoglycemia and adverse outcomes. Current guidelines therefore favor safe and sustainable glycemic control rather than strict normoglycemia [2,4,8]. In this setting, glycemic control depends not only on insulin therapy but also on the carbohydrate load and composition of enteral nutrition.

Standard enteral formulas generally contain more carbohydrate and less fat, whereas diabetes-specific formulas are designed with lower carbohydrate content and higher fat, including monounsaturated fatty acids and fiber. These features may reduce postprandial glycemic excursions, lower insulin requirement, and improve glycemic control. Although some studies have reported favorable glycemic effects with diabetes-specific formulas [9,10,11], these benefits have not been consistently demonstrated across all critically ill populations or glycemic outcomes. In critically ill patients with T2DM, direct comparative data reflecting routine clinical practice remain limited. Moreover, formula selection in the ICU is influenced not only by metabolic goals but also by clinical status, product availability, institutional supply conditions, and cost [2,4].

Accordingly, this retrospective study compared standard and diabetes-specific enteral formulas with respect to glycemic control, insulin requirement, related clinical parameters, and direct product-related cost in critically ill patients with T2DM under pandemic-related product access constraints.

## 2. Materials and Methods

### 2.1. Study Design

This retrospective observational comparative study was conducted in the general intensive care unit of a tertiary training and research hospital. Adult patients with T2DM admitted to the ICU between 21 August 2021 and 21 August 2023 were reviewed retrospectively. The study arose from the need to use standard enteral formulas in some patients because access to diabetes-specific enteral products was restricted during the COVID-19 pandemic.

### 2.2. Ethical Statement

The study was conducted in accordance with the Declaration of Helsinki and approved by the Clinical Research Ethics Committee of Bursa Yüksek İhtisas Training and Research Hospital (approval date: 9 August 2023; protocol number: 2011-KAEK-25 2023/08-20). Informed consent was waived by the Ethics Committee due to the retrospective design of the study and the use of anonymized patient data.

### 2.3. Patient Population

This retrospective study included adult patients with T2DM who received enteral nutrition during ICU stay. Patients were categorized according to the enteral formula used during ICU follow-up as either the standard enteral formula group or the diabetes-specific enteral formula group. To reduce product heterogeneity and improve between-group comparability, the analysis was restricted to patients receiving the most commonly used standard and diabetes-specific formulas. The standard formula group included patients receiving Ensure (1.0 kcal/mL, 250 mL; Abbott Nutrition, Abbott Laboratories, Abbott Park, IL, USA), whereas the diabetes-specific formula group included patients receiving Nutrison Advanced Diason (1.0 kcal/mL, 500 mL; Nutricia Advanced Medical Nutrition, Zoetermeer, The Netherlands). During the COVID-19 pandemic, restricted access to diabetes-specific enteral formulas necessitated the use of standard enteral formulas in some critically ill patients with T2DM. This retrospective study arose from the clinical question of whether standard formulas provided acceptable glycemic and clinical outcomes under these circumstances. In the retrospective screening, all 50 eligible patients who had received a diabetes-specific enteral formula were included. To ensure numerical balance and improve comparability, 50 consecutive eligible patients who had received a standard enteral formula were selected as the comparison group. Enteral formula selection was not randomized and was not determined by investigator preference. During routine clinical care, the administered formula was determined according to product availability in the hospital pharmacy and the patient’s clinical suitability. All included patients received enteral nutrition via continuous infusion through a nasogastric tube. In routine ICU practice, nasogastric tube position was confirmed by chest radiography before enteral feeding initiation. Enteral nutrition was administered as continuous infusion in accordance with routine ICU practice.

### 2.4. Inclusion and Exclusion Criteria

Eligible patients were adults (≥18 years) admitted to the ICU for at least 48 h, with a documented history of T2DM and antidiabetic treatment, no contraindication to enteral nutrition, initiation of nasogastric enteral feeding, receipt of the same enteral formula for at least 7 days, insulin infusion for glycemic control, and at least four daily glucose measurements during this period.

Patients with COVID-19 were excluded because of their potential independent effects on ICU course, metabolic response, and nutritional management. Additional exclusion criteria were age < 18 years, type 1 diabetes mellitus, contraindication to enteral nutrition, enteral feeding through a route other than a nasogastric tube, <7 days of enteral nutrition follow-up, parenteral nutrition, morbid obesity (BMI ≥ 40 kg/m^2^), missing or insufficient data, unclear formula classification, and switching between standard and diabetes-specific formulas during follow-up.

### 2.5. Data Collection

Data were retrospectively obtained from the hospital information system, ICU follow-up charts, nursing observation records, laboratory results, and archived medical records. Collected variables included age, sex, body mass index, reason for ICU admission, comorbidities, APACHE II and SOFA scores, time to enteral nutrition initiation, formula type, hourly infusion volume, total caloric requirement calculated by the nutrition team using the Harris–Benedict equation, daily caloric intake, daily capillary glucose measurements, morning plasma glucose level, laboratory parameters, daily total insulin dose, vasopressor and steroid use, duration of mechanical ventilation, ICU length of stay, complications, and ICU outcome. When available in medical records, pre-admission antidiabetic treatment—including the use of oral antidiabetic agents and/or insulin—was documented. Following admission to the intensive care unit (ICU), previous glucose-lowering therapies were routinely discontinued, and glycemic control was maintained using intravenous insulin infusion in accordance with standard ICU protocols. Since detailed data on specific drug classes, including GLP-1 receptor agonists, were not consistently available for all patients, these data were not included in the analysis.

Recorded laboratory variables included fasting blood glucose, albumin, creatinine, glomerular filtration rate, total bilirubin, white blood cell count, hemoglobin, platelet count, activated partial thromboplastin time, prothrombin time, and international normalized ratio. HbA1c values were not consistently available for all patients and were therefore not included in the analysis. Similarly, diabetic microvascular complications could not be systematically assessed from the retrospective records and were not analyzed as separate variables. Physiological variables included blood pressure, heart rate, baseline fraction of inspired oxygen, peripheral oxygen saturation, arterial oxygen saturation, and lactate level. Recorded complications included hypoglycemia, hyperglycemia, infection, and enteral intolerance. Infection was defined as any clinically or microbiologically documented infection occurring during ICU stay. Enteral intolerance was defined by documented gastrointestinal intolerance, including vomiting, abdominal distension, high gastric residual volume, and/or interruption of feeding.

### 2.6. Glycemic Assessment

Glycemic parameters were assessed using blood glucose measurements recorded in the medical charts during routine clinical follow-up. The frequency of glucose monitoring was determined according to routine ICU clinical protocols and the patient’s clinical condition. Insulin therapy was individualized according to the patient’s clinical condition. In routine ICU practice, capillary blood glucose was measured at least 4–8 times daily, with monitoring increased to as often as hourly in patients receiving insulin infusion.

Hypoglycemia was defined as at least one blood glucose measurement < 70 mg/dL and hyperglycemia as at least one measurement > 180 mg/dL during follow-up. The frequencies of hypoglycemia and hyperglycemia were evaluated as secondary glycemic outcomes. The target glycemic range was defined as 140–180 mg/dL in accordance with current ADA and SCCM recommendations [2,4]. Glycemic control was managed using continuous intravenous insulin infusion according to routine ICU protocols aligned with current guideline recommendations, with infusion rate adjustments based on serial capillary glucose measurements [2,4]. Days on which all recorded minimum and maximum glucose values remained within this range were defined as full control days. For each patient, the total number and proportion of full control days were calculated. Mean glucose range was used as an indicator of glycemic variability. The carbohydrate content of both formulas was obtained from product specifications. Carbohydrate exposure was calculated from the administered enteral volume and the carbohydrate content per 100 mL and was expressed as mean daily carbohydrate intake (g/day).

### 2.7. Cost Assessment

To reduce cost heterogeneity, the analysis was restricted to patients receiving the single standard formula and single diabetes-specific formula most commonly used in hospital practice during the study period. These products were selected based on actual hospital pharmacy availability rather than investigator preference. Direct product-related costs were calculated using hospital pharmacy purchase prices during the study period and included only enteral formula costs; consumables, nursing workload, and other indirect costs were not included.

### 2.8. Study Outcomes

The primary outcomes were mean daily capillary blood glucose level and mean daily insulin requirement during enteral nutrition. Secondary outcomes included glycemic variability, the number and percentage of days within the target glycemic range, the frequency of hypoglycemia and hyperglycemia, infection, ICU length of stay, ICU mortality, and direct product-related cost.

### 2.9. Statistical Analysis

Statistical analyses were performed using IBM SPSS Statistics for macOS, version 30.0 (IBM Corp., Armonk, NY, USA). Categorical variables are presented as n (%), and continuous variables as mean ± SD or median (IQR), as appropriate. Normality was assessed using the Kolmogorov–Smirnov test and skewness/kurtosis values within ±2. Between-group comparisons were performed using Student’s *t*-test or Welch’s *t*-test for normally distributed variables, Mann–Whitney U test for non-normally distributed variables, and chi-square or Fisher’s exact test for categorical variables. A G*Power 3 sensitivity analysis showed that, with a two-sided α = 0.05 and 80% power, 50 patients per group allowed for the detection of a minimum effect size of Cohen’s d = 0.56 [12].

Glycemic variables were derived from daily measurements. Hypoglycemia and hyperglycemia were defined as at least one glucose measurement of <70 mg/dL and >180 mg/dL, respectively. Mean glucose level was calculated as the average of daily glucose measurements, and mean insulin requirement as the average daily total insulin dose. The 7-day mean glucose range, calculated from daily minimum and maximum capillary glucose values, was used as an indicator of glycemic variability. Although standard deviation (SD) and coefficient of variation are more established metrics, they were not used because glucose monitoring frequency and timing varied across patients and continuous glucose monitoring was not available. Therefore, glucose range was interpreted as a pragmatic indicator of glycemic fluctuation rather than a standardized glycemic variability metric. The target glycemic range was defined as 140–180 mg/dL; days on which both minimum and maximum glucose values remained within this range were defined as full control days, and the total number and percentage of such days were calculated. The daily caloric ratio was calculated as delivered calories divided by estimated energy requirement, and its 7-day mean was used in the analyses. Carbohydrate exposure was calculated to better characterize the nutritional intervention and was presented descriptively; however, it was not included in the primary multivariable regression models.

Factors associated with mean insulin requirement and glycemic variability were evaluated using univariable and multivariable linear regression analyses. Clinically relevant variables were included in multivariable models regardless of univariable significance. Model fit was assessed using adjusted R^2^. Sensitivity analyses were performed by substituting SOFA for APACHE II and creatinine for GFR. A two-sided *p* < 0.05 was considered statistically significant. Direct product-related costs were calculated using hospital pharmacy purchase prices and expressed as 7-day total cost, daily cost, and cost per 1000 kcal. Cost variables were compared between groups using Student’s *t*-test or the Mann–Whitney U test, as appropriate.

## 3. Results

**Figure 1:**
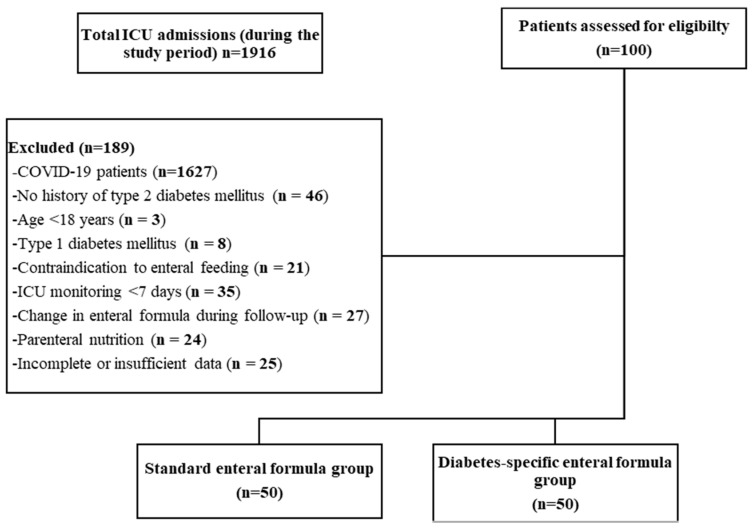
Study flow diagram of patient enrollment and group allocation.

Between 21 August 2021 and 21 August 2023, 1916 patients were admitted to the ICU. Among them, 289 patients without active COVID-19 and with a recorded diagnosis of T2DM were screened for eligibility; 189 were excluded. All eligible patients receiving a diabetes-specific enteral formula were included (n = 50), and 50 consecutive eligible patients receiving a standard enteral formula were selected for comparison. The final analysis comprised 100 patients, with 50 patients in each group (Figure 1).

Baseline demographic and clinical characteristics are presented in Table 1. The groups were comparable in sex, age, APACHE II and SOFA scores, vasopressor use, comorbidity profile, ICU admission diagnosis, time to enteral feeding initiation, and daily caloric requirement (all *p* > 0.05). Body weight showed a borderline difference (*p* = 0.056), whereas BMI was significantly lower in the diabetes-specific formula group (*p* = 0.029). Maximum feeding rate was significantly higher in the diabetes-specific formula group (67.3 ± 11.8 mL/h vs. 60.2 ± 13.1 mL/h, *p* = 0.005). Most admission laboratory and hemodynamic parameters were similar between groups (all *p* > 0.05). However, total bilirubin was higher in the diabetes-specific formula group, whereas heart rate was higher in the standard formula group (*p* = 0.015 and *p* = 0.002, respectively). Overall, baseline differences were limited to BMI, maximum feeding rate, total bilirubin, and heart rate.

### 3.1. Glycemic Outcomes

Findings related to glycemic control, glycemic variability, and insulin requirement are presented in Table 2. Mean glucose level, mean insulin requirement, mean glucose range as an indicator of glycemic variability, the frequency of hypoglycemia and hyperglycemia, and mean caloric ratio were similar between groups (all *p* > 0.05). Mean daily carbohydrate exposure was lower in the diabetes-specific formula group than in the standard formula group (146.0 ± 26.9 vs. 185.4 ± 35.2 g/day). In contrast, the number and percentage of days within the target glycemic range were significantly higher in the diabetes-specific formula group (*p* < 0.05). Hypoglycemia was infrequent and did not differ significantly between groups (4 [8%] in the standard formula group vs. 6 [12%] in the diabetes-specific formula group, *p* = 0.505).

### 3.2. Clinical Outcomes and Complications

Clinical outcomes and complications are presented in Table 3. The groups were similar in terms of duration of mechanical ventilation, ICU length of stay, complications, and ICU outcomes (all *p* > 0.05).

### 3.3. Regression Analyses

Univariable and multivariable linear regression analyses of factors associated with mean insulin requirement are presented in Table 4. In univariable analysis, mean glucose level was positively associated with insulin requirement, whereas vasopressor use was negatively associated (β = 0.373, *p* < 0.001; β = −7.217, *p* = 0.044, respectively). No other variables showed significant associations (*p* > 0.05). In multivariable analysis, only mean glucose level remained independently associated with insulin requirement (β = 0.370, *p* < 0.001). The model explained 30.6% of the variance in insulin requirement (adjusted R^2^ = 0.306).

Univariable and multivariable linear regression analyses of factors associated with glycemic variability are presented in Table 5. Mean glucose level was positively associated with glycemic variability in both univariable and multivariable analyses (β = 0.770 and β = 0.743, respectively; both *p* < 0.001). The model explained 28.9% of the variance in glycemic variability (adjusted R^2^ = 0.289). Sensitivity analyses substituting SOFA for APACHE II and creatinine for GFR yielded similar results, supporting the robustness of the findings.

### 3.4. Cost Analysis

In the cost analysis, total caloric intake over 7 days was similar between groups (*p* = 0.159). However, the total number of bottles and the total, daily, and per-1000-kcal costs were significantly lower in the diabetes-specific enteral formula group than in the standard formula group (all *p* < 0.001) (Table 6).

## 4. Discussion

In critically ill patients with T2DM, the diabetes-specific enteral formula did not provide clear superiority in mean glucose level, insulin requirement, glycemic variability, or short-term clinical outcomes. However, the number and percentage of days within the target glycemic range were higher in this group. A key finding of the study was that the two groups were largely comparable in baseline demographic and clinical characteristics. Multivariable analysis further showed that mean glucose level, rather than formula type, was the main determinant of the observed metabolic outcomes.

In our cohort, the standard and diabetes-specific enteral formula groups were largely comparable in age, sex, APACHE II and SOFA scores, comorbidity burden, ICU admission diagnoses, and baseline glucose levels, supporting baseline comparability in disease severity and overall clinical profile. Similar balance in baseline characteristics was also reported by Mesejo et al. [11]. However, the higher BMI in the standard formula group and the higher maximum feeding rate in the diabetes-specific formula group suggest that glycemic outcomes may reflect not only formula composition but also underlying metabolic profile and nutritional load. Because maximum feeding rate was considered to reflect a similar nutritional burden and to provide overlapping information, mean caloric ratio was included in the multivariable model instead; enteral formula type was not an independent determinant of insulin requirement or glycemic variability. Likewise, studies comparing volume-based and rate-based enteral feeding have shown that, although achievement of energy and protein targets may differ, hyperglycemia and glycemic variability do not always differ significantly [13]. The ESPEN guideline similarly emphasizes that timing, dose, route, and composition of nutrition should be evaluated together [14]. Despite differences in total bilirubin and heart rate, the similarity of APACHE II, SOFA, and hemodynamic parameters suggests that these findings more likely reflect nonspecific physiological heterogeneity accompanying critical illness rather than a true between-group imbalance [15,16].

No significant between-group differences were observed in mean glucose level or mean insulin requirement. Similarly, van Steen et al. reported no clear difference in glycemic variability with a low-carbohydrate formula [17]. These findings suggest that, in critically ill patients with T2DM, diabetes-specific formulas may not substantially reduce overall glycemic burden or total insulin requirement. This finding may be partly related to the lower mean daily carbohydrate exposure observed in the diabetes-specific enteral formula group. However, the higher number and percentage of days within the target glycemic range in the diabetes-specific formula group suggest that these formulas may facilitate maintenance of clinically acceptable glucose control even without significantly lowering mean glucose levels. Large multicenter data also suggest that the association between glycemic variability and mortality may differ according to diabetic status [18,19]. Because glycemic management in the ICU aims to maintain a safe target range without inducing hypoglycemia, this finding may still be clinically relevant. Feeding strategies and glucose monitoring frequency may significantly influence glycemic control in critically ill patients, and optimization of these factors may reduce hypoglycemia and glycemic variability [14]. It is also consistent with review data indicating that low-carbohydrate, high-fat enteral formulas may improve some aspects of glycemic control, although not all glycemic outcomes consistently [20]. In this study, the term “low-carbohydrate” refers to the relative manufacturer-defined macronutrient composition of the diabetes-specific formula rather than to a quantitatively measured daily carbohydrate exposure. Accordingly, the present findings should be interpreted as an exploratory real-world comparison between enteral formula types rather than as evidence of a predefined low-carbohydrate nutritional strategy. In addition, hypoglycemia, a clinically important adverse glycemic outcome in critically ill patients, was infrequent and comparable between groups in our cohort.

Although diabetes-specific enteral formulas may support maintenance of the target glycemic range, this effect appears modest, multifactorial, and should be interpreted cautiously. Despite similar caloric adequacy between groups, the limited sample size may have reduced the ability to detect small-to-moderate differences. Moreover, glycemic control is influenced not only by formula type but also by feeding rate, caloric load, infection, steroid exposure, vasopressor use, organ dysfunction, insulin strategy, and differences in glucose monitoring and reporting [14,19,21]. Inter-individual differences in glucose monitoring frequency may also have influenced the assessment of glycemic variability and time-in-range outcomes, particularly in a retrospective cohort based on routine clinical measurements. Wewalka et al. likewise found no significant differences in mean glucose, insulin requirement, or glycemic variability between formula types, although the exclusion of diabetic patients limits generalizability [22]. Similarly, López-Gómez et al. reported reduced hyperglycemia with a diabetes-specific formula but no effect on length of stay or mortality [23]. Together, these findings suggest that diabetes-specific formulas may improve selected glycemic indices without consistently translating into broader glycemic or clinical superiority [22,23]. Recent randomized data also indicate that tighter glucose control does not necessarily improve mortality or ICU length of stay [24].

No between-group difference was observed in glycemic variability, and multivariable analysis showed that mean glucose level, rather than formula type, was the independent determinant. APACHE II, BMI, steroid use, vasopressor use, renal function, mean caloric ratio, and invasive mechanical ventilation were not independently associated with glycemic variability. Previous studies have reported inconsistent associations of disease severity, steroid exposure, vasopressor requirement, and nutritional load with glycemic variability [11,19,25]. Although van Hooijdonk et al. reported higher glycemic variability with bolus hydrocortisone [26], this relationship may vary according to steroid agent, dose, mode of administration, and insulin strategy. In our study, steroid use showed only a borderline trend, and no independent association was found for vasopressor use, likely reflecting the multifactorial nature of glycemic variability [27]. Overall, glycemic variability appeared to be more closely related to glycemic burden than to formula type. Likewise, mean glucose level, but not formula type, independently predicted insulin requirement, consistent with the absence of a between-group difference in insulin use and suggesting that diabetes-specific formulas may not uniformly reduce insulin needs in all critically ill patients with T2DM [11,19,25,26,27].

No clear superiority of diabetes-specific formulas was observed in short-term clinical outcomes. Although some components of glycemic control appeared more favorable, this did not translate into differences in mechanical ventilation duration, ICU length of stay, infection, or mortality. These outcomes are likely influenced by multiple factors beyond glycemic control, including disease severity, infectious burden, hemodynamic status, and concomitant therapies. Previous studies have linked hyperglycemia to longer mechanical ventilation, but improved glycemic control has not consistently reduced ventilation duration [28,29]. Enteral intolerance is likewise multifactorial and not solely determined by formula type [30]. Despite baseline differences in FiO_2_ and SaO_2_, similar ventilation rates and durations suggest that this pattern more likely reflects underlying respiratory heterogeneity than a formula effect. No differences were also observed in intolerance, infection, or pressure ulcer development. Recent studies have also suggested that feeding strategy and feeding interruptions may affect glycemic outcomes during enteral nutrition [31,32]. Accordingly, some residual confounding related to enteral feeding practice may still be present in our retrospective analysis.

Taken together, these findings suggest that diabetes-specific enteral formulas may provide a more favorable glycemic profile, particularly by improving maintenance of the target glycemic range, but do not confer clear superiority in mean glucose level, insulin requirement, glycemic variability, or short-term clinical outcomes in critically ill patients with T2DM. Conversely, the absence of a clear disadvantage with standard formulas suggests that diabetes-specific formulas may not be mandatory for every critically ill patient with T2DM. Given considerations such as cost, product availability, and practical feasibility in the ICU, enteral formula selection should be individualized according to glycemic targets, metabolic status, feeding tolerance, clinical needs, and logistical constraints.

Notably, despite similar 7-day caloric intake, direct product-related costs were lower in the diabetes-specific formula group. Although diabetes-specific formulas were initially expected to increase costs, our findings suggest that they did not impose an additional direct economic burden and were, in this cohort, associated with lower costs. This is consistent with the findings of Han et al., who also reported lower ICU and daily costs with diabetes-specific formulas in critically ill patients with T2DM [10].

### Limitations

Our study has several limitations. The study had a retrospective observational design; therefore, its ability to establish causal relationships is limited. It was based on retrospective data obtained during a period of unique clinical circumstances, in which access to diabetes-specific enteral formulas was restricted because of COVID-19 pandemic-related supply constraints. Although this confers real-world value to the study, the retrospective group allocation and non-randomized choice of enteral formula may have influenced patient selection and product allocation, thereby increasing the possibility of selection bias. Because the number of eligible patients in the diabetes-specific enteral formula group was limited, the group receiving standard enteral products was selected consecutively and compared using an equal sample size. Although this approach aimed to improve comparability, it may not fully represent the broader population of patients receiving standard enteral nutrition. The sample size may have been insufficient to detect small or moderate effects, particularly with respect to mortality, infection, and other clinical outcomes. Therefore, negative findings should be interpreted cautiously rather than as definitive evidence of equivalence or absence of effect.

Glycemic outcomes may be influenced by disease severity, infection burden, corticosteroid and vasopressor use, organ dysfunction, insulin strategies, and daily nutritional practices in addition to enteral formula type. Although clinically relevant variables were included in multivariable and sensitivity analyses, unmeasured residual confounding cannot be excluded. Detailed standardized insulin adjustment algorithms were not consistently available in the retrospective records; therefore, the potential influence of inter-individual differences in insulin administration cannot be completely excluded. Although enteral nutrition was administered as continuous infusion, short-term interruptions and rate adjustments could not be fully standardized or analyzed because of the retrospective design. The study population was at increased risk for gastroparesis, gastrointestinal dysmotility, and enteral feeding intolerance because of the coexistence of T2DM and critical illness. Although no significant between-group difference in enteral intolerance was observed, this outcome should be interpreted in light of concomitant factors such as critical illness, sedation, vasopressor use, organ dysfunction, and the retrospective nature of intolerance documentation.

Glycemic assessment was based on intermittent capillary and/or plasma glucose measurements recorded in routine clinical practice rather than continuous glucose monitoring (CGM). Therefore, inter-group differences in monitoring frequency may have introduced measurement bias, and short-term glycemic fluctuations or true glycemic variability may have been underestimated. Although general information on pre-admission antidiabetic therapy was available, detailed data on specific drug subclasses, including GLP-1 receptor agonists, were not consistently available for all patients. Therefore, the potential effects of prior diabetes therapy on feeding tolerance and glycemic response could not be fully assessed. In addition, HbA1c values and standardized information on diabetic microvascular complications, such as neuropathy, retinopathy, and nephropathy, were not consistently available in the retrospective records. Accordingly, the potential influence of long-term glycemic control and chronic diabetic complications on the observed outcomes could not be systematically evaluated. Because HbA1c data were incompletely available and enteral formula selection was not randomized, baseline glycemic status may not have been fully comparable between groups despite broadly similar admission glucose levels.

In addition, the cost analysis included only product-based direct costs and did not assess indirect care costs or total intensive care unit expenditures. Therefore, the economic findings should be interpreted as a product-based direct cost comparison rather than a comprehensive health economic evaluation. Detailed quantitative macronutrient delivery, including daily carbohydrate, fat, and protein intake, could not be calculated from the retrospective records; therefore, the findings should be interpreted as a formula-type comparison rather than a precise macronutrient-dose comparison.

Nevertheless, the substantial similarity of baseline characteristics between the groups, the use of data derived from daily clinical practice, and the support of glycemic outcomes by multivariable analyses represent important strengths of the study.

## 5. Conclusions

In critically ill patients with T2DM, diabetes-specific enteral formula showed no clear overall glycemic or clinical superiority over standard formula, although it was associated with more time within the target glycemic range and no additional cost burden. These findings support individualized rather than routine use of diabetes-specific formulas. Prospective randomized multicenter studies are needed to eliminate allocation bias, confirm these findings, and identify the patient subgroups most likely to benefit.

## Figures and Tables

**Table 1:** Baseline demographic and clinical characteristics of the study population.

Characteristics	Standard Enteral Formula Group (n = 50)	Diabetes-Specific Enteral Formula Group (n = 50)	*p*-Value
Sex			0.685
Male	28 (56)	30 (60)	
Female	22 (44)	20 (40)	
Age (years)	71.3 ± 10.3	73.6 ± 9.6	0.259
Weight (kg)	80 (70–85)	70.5 (65–84)	0.056
BMI (kg/m^2^)	27.6 (25.1–32.2)	25.4 (22.5–29.4)	0.029
APACHE II	24.5 ± 4.4	23.7 ± 3.9	0.362
SOFA	6.9 ± 2	7.1 ± 1.8	0.600
Vasopressor	19 (38)	26 (52)	0.159
Comorbidities			
Hypertension	43 (86)	43 (86)	1.000
Coronary Artery Disease	14 (28)	17 (34)	0.517
Congestive Heart Failure	14 (28)	12 (24)	0.648
Arrhythmia	5 (10)	4 (8)	1.000
Other	29 (58)	28 (56)	0.840
Cerebrovascular disease	5 (17.2)	2 (7.1)	0.423
Malignancy	6 (20.7)	2 (7.1)	0.253
Neurologic disease	6 (20.7)	8 (28.6)	0.490
Pulmonary disease	7 (24.1)	11 (39.3)	0.219
Renal disease	5 (17.2)	5 (17.9)	1.000
ICU admission diagnosis			
Cardiovascular pathology	8 (16)	4 (8)	0.218
Cardiovascular system disease	0 (0)	1 (2)	1.000
Cerebrovascular pathology	11 (22)	16 (32)	0.260
Non-diabetic neurological disease	1 (2)	2 (4)	1.000
Pulmonary pathology	19 (38)	19 (38)	1.000
Renal pathology	6 (12)	2 (4)	0.269
Sepsis/Infection	2 (4)	2 (4)	1.000
Trauma	3 (6)	4 (8)	1.000
Time to enteral feeding initiation (days)	1 (1–2)	2 (1–2)	0.176
Maximum feeding rate (mL/hour)	60.2 ± 13.1	67.3 ± 11.8	0.005
Harris–Benedict (kcal/day)	1725 (1500–1900)	1700 (1500–1800)	0.368
Laboratory parameters at admission			
Fasting glucose (mg/dL)	203.1 ± 71	222.3 ± 81.3	0.212
Albumin (g/L)	26.4 ± 5.4	28.8 ± 6.6	0.052
Creatinine (mg/dL)	1.2 (0.8–2.2)	1.4 (0.9–2.3)	0.517
GFR (mL/min/1.73 m^2^)	56.2 ± 33.5	54.8 ± 33.1	0.838
Total bilirubin	0.5 (0.4–0.6)	0.6 (0.4–0.9)	0.015
WBC (×10^3^)	13.1 (10.0–15.8)	12.0 (9.6–14.3)	0.424
Hemoglobin (g/dL)	10 ± 2	10.4 ± 2.3	0.328
PLT (×10^3^)	251.8 ± 119.3	260.8 ± 115.7	0.702
aPTT (seconds)	25.9 (22–29.3)	24.5 (20.1–30.5)	0.268
PT (seconds)	14.3 (13.5–16.1)	13.7 (12.5–14.8)	0.067
INR	1.2 (1–1.3)	1.1 (1–1.5)	0.767
Systolic BP (mmHg)	120.2 ± 22.1	117.6 ± 23.6	0.568
Diastolic BP (mmHg)	67.9 ± 12.3	67.9 ± 15.9	0.994
Heart rate (beats/min)	103.4 ± 19.6	90.8 ± 20.7	0.002

Categorical variables are presented as n (%). Continuous variables are expressed as mean ± standard deviation (SD) for normally distributed data and median (interquartile range, IQR) for non-normally distributed data. BMI: body mass index; APACHE II: Acute Physiology and Chronic Health Evaluation II; SOFA: Sequential Organ Failure Assessment; GFR: glomerular filtration rate; WBC: white blood cell count; PLT: platelet count; aPTT: activated partial thromboplastin time; PT: prothrombin time; BP: blood pressure; ICU: intensive care unit.

**Table 2:** Glycemic control and insulin requirements.

Characteristics	Standard Enteral Formula Group (n = 50)	Diabetes-Specific Enteral Formula Group (n = 50)	*p*-Value
Hypoglycemia (blood glucose < 70 mg/dL)	4 (8)	6 (12)	0.505
Hyperglycemia (blood glucose > 180 mg/dL)	43 (86)	44 (88)	0.766
Mean glucose (mg/dL)	165.8 ± 33.7	160.1 ± 18.2	0.298
Mean insulin (IU/day)	25 ± 20.6	20.3 ± 14.4	0.181
Mean glucose range (mg/dL)	79.4 (60.3–115.1)	74.3 (59.6–99)	0.254
Number of days within target range (days)	0 (0–0)	0 (0–1)	0.021
Percentage of days within target range (blood glucose 140–180 mg/dL)	0 (0–0)	0 (0–14.3)	0.021
Mean daily carbohydrate exposure (g/day)	185.4 ± 35.2	146.0 ± 26.9	<0.001
Mean caloric ratio	0.8 ± 0.1	0.8 ± 0.1	0.384

Categorical variables are presented as n (%). Continuous variables are expressed as mean ± standard deviation (SD) for normally distributed data and median (interquartile range, IQR) for non-normally distributed data.

**Table 3:** Clinical outcomes and complications.

Characteristics	Standard Enteral Formula Group (n = 50)	Diabetes-Specific Enteral Formula Group (n = 50)	*p*-Value
MV	42 (84)	42 (84)	1.000
FiO_2_ (%)	68.1 ± 19.2	59.2 ± 19.2	0.023
SpO_2_ (%)	95.2 ± 3.3	96 ± 3.1	0.192
SaO_2_ (%)	80.3 ± 18.2	86.9 ± 14.8	0.050
Lactate (mmol/L)	2 (1.3–3)	1.7 (1.2–2.3)	0.227
Steroid	10 (20)	10 (20)	1.000
MV duration (days)	14.5 ± 12.9	13.5 ± 11.6	0.685
Intolerance	14 (28)	16 (32)	0.663
Infection	16 (32)	22 (44)	0.216
Pressure ulcer	1 (2)	5 (10)	0.204
ICU length of stay (days)	16.9 ± 11.5	17.5 ± 10.6	0.793
ICU outcome			0.841
Discharge	26 (52)	27 (54)	
Mortality	24 (48)	23 (46)	

Categorical variables are presented as n (%). Continuous variables are expressed as mean ± standard deviation (SD) for normally distributed data and median (interquartile range, IQR) for non-normally distributed data. MV: mechanical ventilation; ICU: intensive care unit; FiO_2_: fraction of inspired oxygen; SpO_2_: peripheral oxygen saturation; SaO_2_: arterial oxygen saturation.

**Table 4:** Univariable and multivariable linear regression analyses of factors associated with mean daily insulin requirement.

Variables	Univariate		Multivariate	
	β (95% CI)	*p*-Value	β (95% CI)	*p*-Value
Group	−4.792 (−11.847–2.264)	0.181	−1.055 (−7.250–5.141)	0.736
Mean glucose (mg/dL)	0.373 (0.264–0.482)	<0.001	0.370 (0.256–0.484)	<0.001
APACHE II	−0.230 (−1.092–0.631)	0.597	0.104 (−0.660–0.868)	0.787
BMI (kg/m^2^)	0.312 (−0.196–0.821)	0.226	0.283 (−0.169–0.735)	0.216
Steroid	3.060 (−5.820–11.939)	0.496	2.658 (−4.902–10.219)	0.487
Vasopressor	−7.217 (−14.225–−0.209)	0.044	−1.915 (−8.460–4.630)	0.563
GFR (mL/min/1.73 m^2^)	0.032 (−0.076–0.140)	0.555	0.039 (−0.057–0.135)	0.425
Mean caloric ratio	15.951 (−12.319–44.221)	0.266	15.139 (−9.218–39.495)	0.220
MV	4.221 (−5.453–13.896)	0.389	1.404 (−7.047–9.855)	0.742

APACHE II: Acute Physiology and Chronic Health Evaluation II; BMI: body mass index; GFR: glomerular filtration rate; MV: mechanical ventilation. Linear regression analysis was performed. Variables considered clinically relevant were included in the multivariable model regardless of univariate significance. Group was coded as 0 = standard enteral formula and 1 = diabetes-specific enteral formula. Steroid use, vasopressor use, and mechanical ventilation were coded as 0 = no and 1 = yes. Continuous variables were entered into the model without transformation. β coefficients represent unstandardized coefficients. The model explained 30.6% of the variance (adjusted R^2^ = 0.306).

**Table 5:** Univariable and multivariable linear regression analysis of factors associated with glycemic variability.

Variables	Univariate		Multivariate	
	β (95% CI)	*p*-Value	β (95% CI)	*p*-Value
Group	−11.432 (−26.502–3.638)	0.135	−7.370 (−20.796–6.056)	0.278
Mean glucose	0.770 (0.533–1.007)	<0.001	0.743 (0.496–0.990)	<0.001
APACHE II	−1.142 (−2.974–0.691)	0.219	−0.908 (−2.564–0.748)	0.279
BMI	0.140 (−0.956–1.236)	0.800	0.014 (−0.965–0.993)	0.977
Steroid	16.173 (−2.604–34.949)	0.091	15.107 (−1.278–31.491)	0.070
Vasopressor	−8.680 (−23.902–6.541)	0.261	0.835 (−13.349–15.020)	0.907
GFR	−0.060 (−0.291–0.171)	0.606	−0.067 (−0.276–0.142)	0.524
Mean caloric ratio	24.398 (−36.313–85.110)	0.427	31.393 (−21.392–84.18)	0.241
MV	9.330 (−11.376–30.036)	0.373	1.390 (−16.925–19.705)	0.880

APACHE II: Acute Physiology and Chronic Health, BMI: body mass index, GFR: glomerular filtration rate, MV: mechanical ventilation. Linear regression analysis was performed. Variables considered clinically relevant were included in the multivariable model regardless of univariate significance. Group was coded as 0 = standard enteral formula and 1 = diabetes-specific enteral formula. Steroid use, vasopressor use, and invasive mechanical ventilation were coded as 0 = no and 1 = yes. Continuous variables were entered into the model without transformation. The model explained 28.9% of the variance (adjusted R^2^ = 0.289).

**Table 6:** Cost analysis of standard and diabetes-specific enteral formulas.

Variable	Standard Enteral Formula Group (n = 50)	Diabetes-Specific Enteral Formula Group (n = 50)	*p*-Value
Total caloric intake over 7 days, kcal	9540.6 ± 1813.7	9045.0 ± 1667.5	0.159
Number of bottles used over 7 days, median (IQR)	40.0 (34.25–43.75)	18.0 (17.0–21.0)	<0.001
Total cost over 7 days, USD, median (IQR)	61.68 (52.81–67.47)	40.43 (38.18–47.17)	<0.001
Daily cost, USD/day, median (IQR)	8.81 (7.54–9.64)	5.78 (5.45–6.74)	<0.001
Cost per 1000 kcal, USD/1000 kcal, median (IQR)	6.26 (6.21–6.30)	4.60 (4.51–4.65)	<0.001

Values are presented as mean ± standard deviation or median (interquartile range [IQR]), as appropriate. Product-related direct costs were calculated using hospital pharmacy purchase prices valid during the chart review period. Total bottle number was calculated by dividing the 7-day administered volume by the package volume of the corresponding formula and rounding up to the nearest whole bottle, since each opened bottle was considered a full-cost unit regardless of partial use. Total administered volume was derived from total caloric intake, assuming an energy density of 1.0 kcal/mL for both formulas.

## Data Availability

The data supporting the findings of this study are available from the corresponding author upon reasonable request, subject to ethical and institutional restrictions.

## Abbreviations

The following abbreviations are used in this manuscript:

T2DM	Type 2 Diabetes Mellitus
APACHE II	Acute Physiology and Chronic Health Evaluation II
SOFA	Sequential Organ Failure Assessment
ADA	American Diabetes Association
SCCM	Society of Critical Care Medicine
BMI	Body Mass Index
GFR	Glomerular Filtration Rate
aPTT	Activated Partial Thromboplastin Time
PT	Prothrombin Time
INR	International Normalized Ratio
FiO_2_	Fraction of Inspired Oxygen
SpO_2_	Peripheral Oxygen Saturation
SaO_2_	Arterial Oxygen Saturation
WBC	White Blood Cell Count
BP	Blood Pressure
ICU	Intensive Care Unit
MV	Mechanical ventilation
ESPEN	European Society for Clinical Nutrition and Metabolism
